# Comparison of early surgical outcomes of robotic and laparoscopic colorectal cancer resection reported by a busy district general hospital in England

**DOI:** 10.1038/s41598-024-57110-1

**Published:** 2024-04-22

**Authors:** Valentin Butnari, Momotaz Sultana, Ahmer Mansuri, Christopher Rao, Sandeep Kaul, Richard Boulton, Joseph Huang, Nirooshun Rajendran

**Affiliations:** 1 Colorectal Department, Barking, Havering and Redbridge University NHS Trust, London, UK; 2 Colorectal Department, North Cumbria Integrated Care NHS Foundation Trust, Cumberland Infirmary, Carlisle, Cumbria, UK; 3 Blizard Institute, Barts and the London School of Medicine & Dentistry Queen Mary, University of London, London, United Kingdom

**Keywords:** Surgical oncology, Outcomes research

## Abstract

Robotic platforms provide a stable tool with high-definition views and improved ergonomics compared to laparoscopic approaches. The aim of this retrospective study was to compare the intra- and short-term postoperative results of oncological resections performed robotically (RCR) and laparoscopically (LCR) at a single centre. Between February 2020 and October 2022, retrospective data on RCR were compared to LCR undertaken during the same period. Parameters compared include total operative time, length of stay (LOS), re-admission rates, 30-day morbidity. 100 RCR and 112 LCR satisfied inclusion criteria. There was no difference between the two group’s demographic and tumour characteristics. Overall, median operative time was shorter in LCR group [200 vs. 247.5 min, *p* < 0.005], but this advantage was not observed with pelvic and muti-quadrant resections. There was no difference in the rate of conversion [5(5%) vs. 5(4.5%), *p* > 0.95]. With respect to perioperative outcomes, there was no difference in the overall morbidity, or mortality between RCR and LCR, in particular requirement for blood transfusion [3(3%) vs. 5(4.5%), *p* 0.72], prolonged ileus [9(9%) vs. 15(13.2%), *p* 0.38], surgical site infections [5(4%) vs. 5(4.4%), *p* > 0.95], anastomotic leak [7(7%) vs. 5(4.4%), *p* 0.55], and re-operation rate [9(9%) vs. 7(6.3%), *p* 0.6]. RCR had shorter LOS by one night, but this did not reach statistical significance. No difference was observed in completeness of resection but there was a statically significant increase in lymph node harvest in the robotic series. Robotic approach to oncological colorectal resections is safe, with comparable intra- and peri-operative morbidity and mortality to laparoscopic surgery.

## Introduction

Despite the falling incidence rates of colorectal cancer due to national screening programmes, colorectal cancer remains the third most common cancer in both sexes, accounting for 10% of all malignant disease and conspicuously holding second rank in global cancer-related deaths at 9.4%^1^.

While neo-adjuvant chemoradiotherapy has afforded 20–35% of patients with rectal cancer a complete pathological response^2^ complimented with a judicious ‘watch and wait’ policy^3,4^, resectional surgery remains the curative treatment modality of choice for colon and rectal cancer. The last few decades have seen a shift from conventional open access surgery to advanced laparoscopic techniques owed to the benefit of reduced tissue trauma, attenuated stress response, reduced post-operative pain, early ambulation, shorter in-patient stay and a more desirable cosmesis, without compromising long-term oncological outcomes. As such, minimally invasive surgery is now considered the gold-standard approach. However, laparoscopic techniques are reliant on the experience of the assistant and are plagued by unstable images and difficulty in achieving good, sustained exposure, especially in the confined space of the pelvis.

The prohibitive cost of robotic surgery without the demonstratable benefits in early literature^5^ has hindered its widespread up-take, but a renaissance in robotic surgery has driven technological advancements in robotic systems, which has increasingly become an evolved alternative minimally invasive option. This is reflected in the exponential rise in publications on this topic in the field of colorectal surgery and other specialities impeded by difficult anatomical access. Despite the lack of haptic feedback, the robotic approach has repeatedly been shown to be safe and non-inferior to laparoscopic surgery^5^. There are, however, some clear advantages to robotic surgery: the platform provides a mechanism to stabilise tremor, the high-definition self-controlled camera provides three-dimensional views, and the articulating instruments improve ergonomics and dexterity of movements. Taken together, these features enhance the ability to perform precise dissection in a narrow surgical field and reduce operator fatigue and strain injuries^6–8^. Consequently, robotic platforms are more often used in performing rectal rather than colonic surgery. With increased global experience and the arrival of the DaVinci Xi, surgeons have begun right^9,10^ and multi-quadrant surgery for both benign and malignant disease.

With the latter, improved short-term outcomes in high BMI, high-risk patients with advanced disease, lower conversion rates and improved circumferential resection margin have been reported^11^. All studies being performed in tertiary centres with high level of expertise. However, evidence supporting the use of robotic platform in treatment of CRC in smaller centres is limited particularly in district general hospitals. To date, and as far as authors are aware this study represents the largest case series performed within 2 years in a district general hospital in England presented in the literature.

### Aims

To evaluate the implementation phase of new service in our district general hospital we designed a retrospective study to compare intra-operative and short-term post-operative results of cancer resections performed robotically (RCR) to established laparoscopic colorectal cancer resections (LCR) over the same period of time.

## Materials and methods

### Study design

We retrospectively compared robotic and laparoscopic colorectal procedures, performed between February 2020 and October 2022 at a single tertiary high-volume centre, performing over 250 cancer resections annually.

Surgeons’ robotic training and competency were individually verified by proctorship. All candidates suitable for a minimally invasive approach were considered for robotic surgery. With collective experience exceeding 20 cases, all subsequent procedures were preferentially treated robotically unless this platform was unavailable. All surgeries were performed by three experienced consultant laparoscopic surgeons.

### Inclusion and exclusion criteria

Selection criteria for RCR centred on factors conducive to minimal access surgery, not exhaustive of body mass index (BMI) ≤ 35, a non-hostile abdomen and adequate physiological reserve for a sustained pneumoperitoneum. Only confirmed cancers of the colorectum, and lesions with suspicious histopathological features without conclusive invasion, but incongruent radiological features for benign disease were considered for analysis. All malignancies were primary and only resections with curative intent have been included. Emergent, palliative, beyond TME (except for isolated pre-sacral fascial dissection), exenterative and those requiring simultaneous non-pelvic visceral/organ resections were excluded. As were open, laparo-endoscopic procedures.

### Variables

Patient demographics: Age, Sex, BMI, American Society for Anaesthesiology (ASA) grading and World Health Organisation (WHO) performance status were collected from patients’ notes and electronical medical records. Tumour characteristics and intraoperative parameters identified for comparison included surgical procedure, stoma formation, access time (from first skin incision to completion of robotic docking), console time (from completing docking to commencement of extraction site), total operative time (TOT: from first skin incision until suturing of last incision) and conversion (open extension of the initially planned incision and/or switching to laparoscopic approach from robotic commencement). Outcome measures include 30-day post-operative complications conferring to the Clavien-Dindo classification. Anastomotic leaks were considered in cases of clinical or radiological features of anastomotic dehiscence. Prolonged ileus was defined as an ileus exceeding four days duration. Requirement for transfusion was used as a surrogate marker of significant blood loss, as this was inconsistently documented, or marked as negligible. Length of in-patient stay (LOS) measured in nights, re-admission rates and 90-day mortality was also recorded. Specimen quality, measured by completeness of resection and lymph node yield is presented.

### Pre-operative

All patients underwent a standard preoperative workup and discussion in the local colorectal cancer multi-disciplinary meeting. Mechanical bowel preparation and oral antibiotics were given for all left-sided procedures while oral antibiotics alone was prescribed for all right and semi-obstructive left-sided lesions.

### Surgical technique

For all left-sided resections, a modified Lloyd-Davies position was adopted, with 23 degrees head down for RCR and more extreme cephalid dependence for LCR. For right-sided resection, patients were positioned supine or in Lloyd-Davis as per surgeons’ preference and the extent of intended lymphadenectomy. For RCR, pneumoperitoneum was achieved via Veress needle insufflation or open access via intended stoma or extraction site. Open Hassan technique was used to establish pneumoperitoneum in all laparoscopic cases. Standard medial to lateral dissection respecting the avascular embryological planes and high pedicle division between haemolocks was performed for all resections. All anastomoses were stapled. Left-sided anastomosis were fashioned end-to-end with 29 mm intra-luminal circular stapler (Touchstone International Medical Science Co. Ltd, Suzhou, China). Most right-sided anastomoses were performed extra-corporally as a standard Barcelona with PROXIMATE® TLC75 linear stapler (Ethicon). Seven right-sided RCR were performed intracorporeally as iso-peristaltic, using SureForm 60 linear stapler (Intuitive Surgical, Inc., Sunnyvale CA, USA) and robotic-oversew of enterostomy. Specimen extraction was via Pfannenstiel incision, extension of midline port or through intended stoma site.

**Figure 1:**
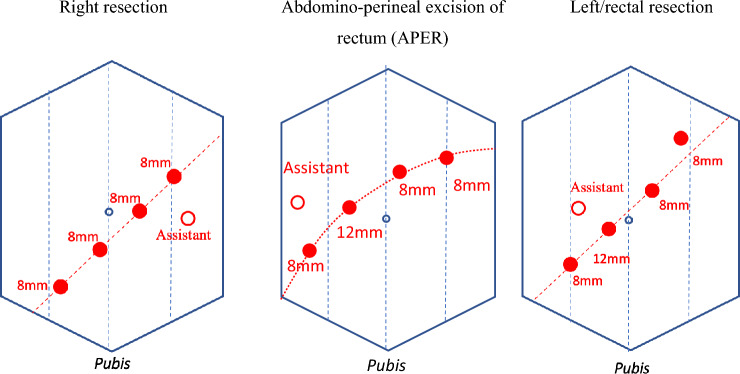
Standard Robotic port configuration.

All robotic resections were performed using the single console DaVinci Xi (Intuitive Surgical, Inc., Sunnyvale CA, USA). A four-port technique (Fig. 1) with 7–8 cm of separation and an additional 12 mm assistant port (Airseal ®, Applied Medical, USA) was used for all resections, as was the two right and one left-handed instrument configuration.

### Post-operative management

Post-operative instructions applied for both LCR and RCR unless otherwise contraindicated, was via the ERAS protocol^12^. Nasogastric tubes were removed prior to anaesthetic reversal, while peritoneal drains (rarely present) and Foley catheters were withdrawn on postoperative day one. Anticoagulation for venous thromboembolism prophylaxis was started 6 h after surgery. Early ambulation and a non-restrictive diet were encouraged from post-operative day one. Discharge was dependant on adequate pain control with oral analgesia, bowels opening, mobility and stoma independence.

### Statistical analysis

All statistical analysis was performed using GraphPad Prism (Version 9.4.1, GraphPad Prism Software, U.S.A). Continuous data is presented as mean and standard deviation, and differences tested using Student’s t-test, unless of a non-Gaussian distribution. Test of proportions was assessed using Fisher's exact test unless otherwise stated, with p value set at 0.05 significance.

### Ethical approval

Barking, Havering and Redbridge NHS Trust research ethics committee exempted this study from ethics approval and informed consent due to the fact that this study was an audit and no patient identifiable data was used. This study follows institutional guidelines on information and research governance. This study is registered with trust audit and research department with the unique identifying number L-227-22. This study has been reported in line with the ‘Strengthening the reporting of cohort studies in surgery’ (STROCSS) criteria^13^.

### Informed consent

Barking, Havering and Redbridge NHS Trust research ethics committee exempted this study from ethics approval and informed consent due to the fact that this study was an audit and no patient identifiable data was used. This study follows institutional guidelines on information and research governance. This study is registered with trust audit and research department with the unique identifying number L-227-22.

## Results

**Figure 2:**
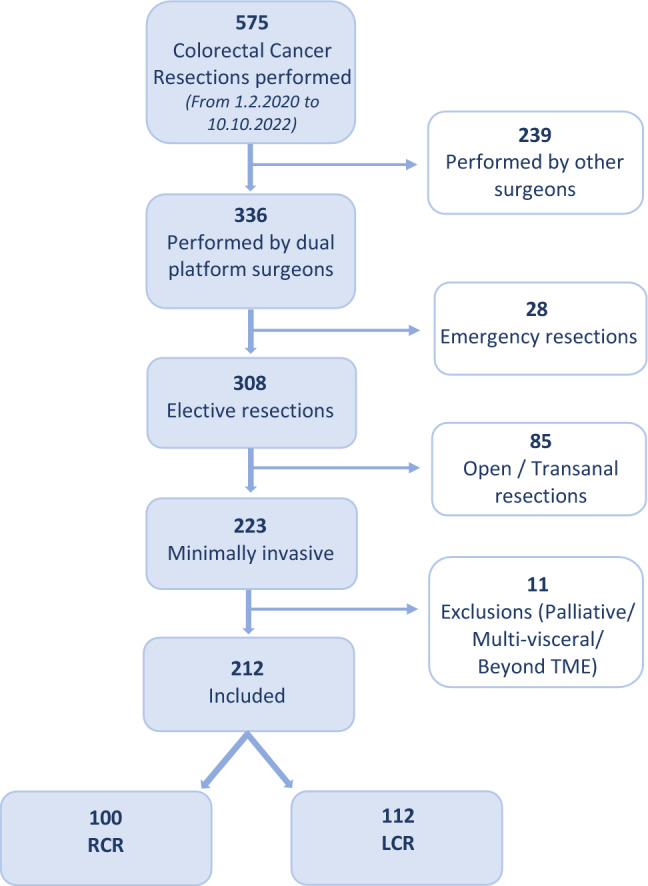
Flow diagram of included cases.

Between February 2020 and October 2022, a total of 308 resections for malignant disease of the colon and rectum were performed electively or semi-electively by the designated surgeons (Fig. 2). 96 cases did not satisfy the inclusion criteria and thus data is presented on the remaining 112 LCR and 100 RCR consecutive cases. The study period incorporated both UK Covid-19 isolation phases, reflecting the reduced operative numbers indicated.

### Demographic Data

The demographic data for each group is presented in Table 1. There was no difference in the age (*p* 0.12), sex (*p* 0.67), ethnicity (*p* > 0.95), or peri-operative risk profile of patients in the LCR and RCR groups, as measured by previous abdominal surgery (*p* 0.17), BMI (*p* 0.55), ASA (*p* 0.55) and WHO (*p* 0.48) performance status.

**Table 1:** Demographic data and presentation.

	RCR n (%)	LCR n (%)	p
n	100	112	
M/F	56 (56)/44 (44)	59 (52.67)/53 (47.32)	0.67
Age	Med 68 [IQR 57–74.6]	Med 71 [IQR 62–75.6]	0.12
Ethnicity			
White British	84 (84)	93 (83.03)	>0.95
Other	16 (16)	19 (16.96)	
BMI	Med 27.2 [IQR 24.2–30.2]	Med 28 [IQR 24.1–31.8]	0.55
Previous abdomino-pelvic surgery	19 (19)	13 (11.60)	0.17
Anticoagulation (Warfarin/DOAC)	9 (9)	17 (15.17)	0.2
ASA			0.55
1	3 (3)	4 (3.57 )	
2	68 (68)	69 (61.60)	
3	29 (29)	32 (28.57)	
4	0	2 (1.78)	
WHO			0.48
0	52 (52)	63 (56.25)	
1	26 (26)	39 (34.82)	
2	12 (12)	10 (8.92)	
Presentation			0.03
Symptomatic expediated referral	66 (66)	81(72.32)	
National bowel cancer screening	15 (15)	26 (23.21)	
Surveillance (CRC/IBD)	6 (6)	2 (1.78)	
Emergency	6 (6)	3 (2.67)	
Other	5 (5)	0	

### Presentation

Symptomatic patients in both cohorts were predominantly referred by their primary healthcare provider, via the expedited cancer pathway. A statistically higher proportion of patients attended as emergencies in the RCR group, as symptomatic anaemia or overt lower-gastrointestinal bleed. Acute appendicitis accounted for one emergency presentation in the RCR arm and both emergency presentations in the LCR group. All patients with impending obstruction were defunctioned prior to definitive planned treatment. Those requiring emergency resection have been excluded from analysis.

Collectively eight patients had a history of malignant disease of the colorectum; six were diagnosed with a non-anastomotic metachronous tumour during their 5-year surveillance period, one had a complete pathological response to neoadjuvant chemoradiotherapy three years previously and had entered surveillance without surgery and one patient developed a new tumour 10 years after the initial primary. Two patients in the RCR cohort had malignant disease detected during surveillance for inflammatory bowel disease (IBD).

### Tumour site and characteristics

104 cancers were present in 100 individuals in the RCR cohort, accounting for four cases with synchronous tumours. Two of these patients required dual resection, while the proximity of the cancers in the other two permitted a single resection. There were no synchronous tumours in the LCR group. Tumour characteristics are outlined in Table 2. There were no statistical difference in tumour distribution (*p* 0.44), grade (*p* 0.72), clinical stage (*p* 0.5), histology (*p* 0.58), or size (*p* 0.69), between the RCR and LCR groups.

**Table 2:** Tumour characteristics.

Tumour	RCR n (%)	LCR n (%)	p*
n	100 (%)	112(%)	
Location			
Right colon (appendix, AC, HF, TC)	50 (48.07)	50 (44.64)	0.44
Left colon (SF, DC, Sig)	37 (35.57)	36 (32.14)	
Rectum	17 (16.34)	26 (23.21)	
Height from AV (cm)			0.72
Low (<5cm)	5 (29.41)	7 (26.92)	
Mid (5-10)	7 (41.17)	14 (53.84)	
High (10-15)	5 (29.41)	5 (19.23)	
Neoadjuvant Chemoradiotherapy	7 (41.17)	16 (61.53)	0.33
Pre-operative histology			
Un-confirmed invasive disease	17 (16.34)	6 (5.35)	<0.05
Adenocarcinoma			0.58
Standard	82 (78.84)	98 (87.50)	
Mucinous/Goblet/Signet	3 (2.88)	7 (6.25)	
Other (NET/ LAMN/Carcinoid)	2 (1.92)	1 (0.89)	
Differentiation			0.72
Well	5 (5.74)	9 (8.49)	
Mod	78 (89.65)	89 (83.9)	
Poor/anaplastic	4 (4.59)	4 (3.77)	
cTNM			
T			
T1/T2	33 (31.73)	38 (33.92)	0.65
T3	56 (53.84)	55 (49.10)	
T4	15 (14.42)	19 (16.96)	
N			
N0	46 (44.23)	42 (37.50)	0.44
N1	39 (37.5)	43 (38.39)	
N2	19 (18.26)	27 (24.10)	
M			
M0	95 (91.36)	107 (95.53)	0.26”
M1	9 (8.65)	5 (4.47)	
Clinical AJCC STAGE			
Stage I	26 (25)	26 (23.21)	0.5
Stage II	19 (19)	15 (13.39)	
Stage III	50 (50)	66 (58.92)	
Stage IV	5 (5)	5 (4.46)	
Size (mm)	33.6 ± 14.9 SD	34.6 ± 18.98 SD	0.69^#^

*Chi Sq, Fisher’s exact test; ^#^t-test.

Twenty three patients did not have confirmed invasive disease prior to resection, Seventeen in the RCR and 6 in LCR (*p* < 0.05). Surgery was offered following multi-disciplinary consensus, based on highly suspicious clinical, histological and radiological features, inability to excise endoscopically and individual choice. All within the LCR and 12 in the RCR were subsequently diagnosed with invasive disease on assessment of the resected specimen.

### Operative data

For the purpose of analysis resections were categorised as (1) Right sided, (2) Left sided; encompassing left hemicolectomies (LH), high anterior resections (HAR) and Hartmann’s (HP), (3) Pelvic; comprising low anterior resections (LAR) and abdominoperineal excision of the rectum (APER) and (4) Multi-quadrant; subtotal colectomies (STC), pan-proctocolectomies (PPC) and dual resections. The latter was necessary in two of the four patients with synchronous tumours. Both underwent a right hemicolectomy, with either a HAR or APER. Overall and resection specific comparisons are presented in Tables 3 and 4 respectively.

**Table 3:** Overall outcome.

	RCR (100) n (%)	LCR (112) n (%)	OR [95% CI]	*p*
Procedural				
Overall access and docking time (min)	28 (IQR 21–39)	/		
Overall console time (min)	140 (IQR 86.3–217)	/		
Overall TOT (min)	247.5 (IQR 190–315)	200 (IQR 170–270)		< 0.05
All stoma	15 (15)	18 (16.07)		> 0.95
Conversion to open	5 (5)	5 (4.46)	1.1 [0.35–3.57]	> 0.95
Sphincter preservation in rectal cancers	7 (41.25)	7 (26.93)	1.9 [− 0.17 to 0.45]	0.50
Excised/Discontinuity	6/1	7/1		
Specimen quality				
LN Yield	20 (IQR 16–27.5)	18.5 (IQR 13–25)	2.2 [0.52–12.2]	0.02
R1	4 (4)	2 (1.78)	2.29 [0.52–12.2]	0.40
Tumour size	33.6 ± 14.9	34.6 ± 18.98		0.69^#^
pT0	10 (10)	5 (4.46)		
Complications				
Transfusion requirement	3 (3)	5 (4.46)	0.66 [0.1–2.5]	0.72
SSI	5 (5)	5 (4.46)	1.12 [0.3–3.5]	> 0.95
Prolonged ileus	9 (9)	15 (13.39)	0.63 [0.2–1.5]	0.38
Clavien-Dindo 3–4	13 (13)	9 (8.03)	1.7 [0.6–4.0]	0.20
Anastomotic leak	7 (7)	5 (4.46)	1.6 [0.5–4.5]	0.55
Re-operation	9 (9)	7 (6.25)	1.48 [0.5–3.8]	0.60
Clavien-Dindo 5 / Mortality	2 (2)	3 (2.67)	0.74 [0.1–3.6]	> 0.95
In-patient stay (No. nights)	5 (IQR 3–8)	6 (IQR 4–10.3)		0.09
Re-admission rate	12 (12)	14 (12.50)	0.95 [0.4–2.1]	> 0.95

# 1 patient had dual CME + EXT Rt, IQR, interquartile range.

**Table 4:** Outcomes stratified by resection.

	RCR	LCR	*p*	RCR	LCR	*p*	RCR	LCR	*p*	RCR	LCR	*p*
Resections n (%)	Right 45 (45)	# Right: 51 (45.53) 51 (45.15)#	0.18	Left 35 (35)	Left 28 (25)	0.13	Pelvic 15 (15)	Pelvic 30 (26.78)	0.04	MQR 5 (5)	MQR 3 (2.67)	0.47
n (%)	STD: 34 (75.55)	STD: 34 (66.66)	0.34	LH: 2 (5.71)	LH: 2 (7.14)	0.95	LAR: 11 (73.33)	LAR: 24 (80)	0.70	STC: 2 (40)	STC: 3 (100)	
	CME: 4 (8.88)	CME: 10 (19.60)		HAR: 31 (88.57)	HAR: 24 (85.71)		APER: 4 (26.66)	APER: 6 (20)		PPC: 1 (20)	PPC: 0	
	EXT: 7 (15.55)	EXT: 8 (15.68)		HP:2 (5.71)	HP: 2 (7.14)					Dual Resect: 2 (40)	Dual Resect: 0	
Access & docking time (min)	Med 27 [IQR 20–38]	/		Med 28 [IQR 23–34.5]	/		MED 35 IQR 26–49	/			/	
Console time (min)	Med 112.5 [IQR 69.2–172.5]	/		Med 130 [IQR 90-220)]	/		Med 240 [IQR 113.5–265] 70–290	/		Med 267.5 [IQR 220–315]	/	
TOT (min)	Med 200 [IQR 166.3–273.8]	Med 185 [IQR 155–213.8]	0.03	Med 250 [IQR 210–300]	Med 200 [IQR 166.3–270]	0.007	Med 300 [IQR 260–360]	Med 275 [IQR 192.5–340]	0.21	Med 367 [IQR 255–435]	Med 315 [IQR 270–360]	0.66
Conversion to open n (%)	0 (0)	1 (1.96)	>0.95	4 (11.42)	1 (3.57)	0.37	1 (6.66)	3 (10)	>0.95	0 (0)	0 (0)	
Max Tum size (mm)	33.7 ± 14.1	40.9 ± 21.7	0.09	35.7 ± 14.6	30.2 ± 16	0.17	27.9 ± 16.6	26.1 ± 10.8	0.69	37.5 ± 24.8	48 ± 19.7	0.63
LN Harvest Mean ± SD	22.7 ± 11.7	21.09 ± 7.4	0.45	23.3 ± 10.5	19.4 ± 7.4	0.09	19.1 ± 8.1	17.2 ± 6.7	0.40	49.5 ± 13.2	23.3 ± 7.6	0.02
R1 n (%)	1 (2.22)	0	0.48	0	1 (3.57)	0.45	1 (6.66)	1 (3.33)	0.25	2 (40)	0	0.95
LOS (Days)	Med 5 [IQR 3–7.75]	Med 5 [IQR 4–12]	0.15	Med 5 [IQR 3–6]	Med 5 [IQR 4–9]	0.15	Med 7.5 [IQR 5–12.5]	Med 6 [IQR 5–8.5]	0.48	Med 10.5 [IQR 8.5–17]	Med 30.5 IQR [11–50]	0.40
CD III–V n (%)	6 (13.33)	6 (11.76)	0.53	3 (8.57)	1 (3.57)	0.62	4 (26.66)	5 (16.66)	0.40	3 (60)	1 (33.33)	>0.95

*Inadequate data for meaningful analysis,^#^one Rt hemi CME + EXT, IQR = interquartile range.

More pelvic resections were performed in the laparoscopic group because in our learning curve for robotics high anterior resections and right hemicolectomies were required prior to a TA300 course and learning TME + splenic flexure mobilisation.

Seven of the 45 robotic right resections had intracorporeal anastomoses. Five patients in each group were converted to a laparotomy due to under-staging of tumour or technical difficulties hindering progress (*p* > 0.95). Only one of these patients had previous had abdominal surgery. Stoma formation (*p* > 0.95), neoadjuvant pelvic irradiation (*p* 0.33), and sphincter preservation (*p* 0.50) rates either due to excision or permanent discontinuity, were also comparable between robotic and laparoscopic approach.

There was considerable variability in both the access [Med 28 (IQR 21–39, range 89)] and console time [Med 140 (IQR 86.3–217), range 570] irrespective of the laterality of the resection. There was a significant difference in the overall TOT between the minimally invasive approaches (median 247.5 vs. 200 min, *p* < 0.05) in favour of LCR. This advantage did not hold true when comparing pelvic (*p* 0.21) and multi-quadrant resections (*p* 0.66).

### Oncological parameters

There was no difference in the completeness of resection between RCR and LCR (*p*. 0.04). However, the lymph node retrieval was significantly increased (*p* 0.02) with robotic approach as compared to laparoscopic approach, in particular for multi-quadrant resections. A total of 4 R1 resections were identified among 100 robotic surgery patients. These resections occurred in 3 patients. One patient had synchronous right-sided colon cancer and low rectal cancer. The right-sided specimen was classified as an R1 resection due to a positive lymph node and a positive circumferential margin (0.5 mm posteriorly at site of previous tumour perforation). This synchronous resection was performed early in the learning curve.The other 2 R1 robotic resections were identified in the cohorts of 2 other surgeons. One was a right hemicolectomy that was understaged radiologically. The last R1 resection was in a low rectal resection also performed early in the learning curve. Six (3%) patients did not have malignancy in the resected specimen. One patient had appendiceal LAMN but no residual disease, and five had only suspicious preoperative disease (HGD).

### Complications and convalescence

The overall morbidity (Clavien-Dindo I–IV) and complications requiring intervention or re-operation (Clavien-Dindo III and IV) for RCR and LCR are likewise comparable [10.8% vs. 9.9%, *p* 0.82] (Table 3). Specifically, there were 7 (6.9%) and 5 (4.4%) anastomotic leaks in the RCR and LCR groups respectively (*p* 0.55), all of which required surgery in the RCR cohort, with 100% anastomotic preservation. By contrast, three of the five LCR anastomotic dehiscence required surgery beyond antibiotics and nutritional support.

LOS for RCR was on average one day shorter than LCR, but this did not reach statistical significance (*p* 0.09). Readmission rates however remained similar between both groups (*p* > 0.9).

## Discussion

Robotic surgery has gained significant traction in recent years, particularly in the field of rectal and pelvic surgery, owing to its enhanced visualization, improved articulation, and greater precision and ergonomic advantages over laparoscopic approaches. Systematic reviews suggest robotic TME for rectal cancer has comparable oncological and recovery parameters to laparoscopic, open or TaTME approach^14^ with reduced rates of conversion compared to LCR^15^, akin to the data presented in this series, where no difference in CRM positivity between the two minimally invasive approaches for right, left and rectal lesions was seen.

In addition we are reporting a small but significant increase in lymph node harvest in robotic resections. While the immaturity of our data prevents extrapolation of survival advantage, an increased LN yield is not unique in the literature^9,10^ and longitudinal studies report comparable medium and long-term oncological outcome with RCR^16,17^.

This paper presents our unit's initial experience with utilizing a robotic platform for surgical treatment of colorectal cancer. We report on the first 100 consecutive robotic colorectal cancer resections performed by a robotic team comprising three surgeons. This experience is compared to a cohort of patients representing all laparoscopic resections performed by the same team within the same period. While acknowledging the early phase of adopting this technology, the findings suggest that robotic colorectal surgery is safe, feasible to the established laparoscopic approach. These findings align with other studies reported on robotic colorectal surgery^18–20^. Despite the prolonged surgical times for non-pelvic RCR, RCR was not associated with increased VTE, conversion, peri-operative morbidity, unscheduled re-operation and mortality rates, reflecting the findings of a recent systematic review of randomised control trials comparing laparoscopic and robotic resections^21^. The operative times for pelvic and multi-quadrant resections inclusive of pelvic resections did not require additional operative time, signifying the widely accepted advantage of muti-articulating limbs in pelvic dissection. Based on our experience in the setting of surgical expertise with established laparoscopic approach in daily practice we consider that level of comfort can be reached in 30–50 cases. We noticed form our experience that high volume experience with laparoscopic surgery is a very important factor in reducing the robotic learning curve. Further learning curve studies need to be done to analyse the performance of surgeons who began their practice with robotic surgery coming from an open or laparoscopic surgical background.

In addition, we report a reduction in the average length of in-patient stay by one day. While this did not reach statistical significance, this may potentially offset the cost of increased intra-operative time and disposables associated with robotic surgery. The earlier discharge and reduction in morbidity has been reported by other centres^22,23^. The explanation for reduction in hospital stay is beyond the scope of this study, but is likely to be multifactorial, including patient expectation and reduced post-operative pain leading to early ambulation. It is feasible that unconscious bias in peri-operative management may have influenced earlier discharge, but the analogous re-admission rates between LCR and RCR suggests the clinical appropriateness of discharges. Earlier series report longer in-patient stay and higher rates of admission to high dependency units with robotic resections. While the latter may reflect a true clinical need, over judicious intensivist support when utilising new surgical techniques was observed with the induction of laparoscopic surgery, which may also account for this observation. Increased utilisation of level 2 and 3 post-operative support was not the practice or the experience of this unit.

The inherent bias and limitation of retrospective data analysis is acknowledged by the authors. In addition, we have included all procedures from the introduction of the DaVinci Xi and thus incorporated the learning curve of all three surgeons with the implication of underestimating the potential benefit of the robotic approach. The novelty of robotic surgery is reflected in the high variability in peritoneal access, docking and console time reported. Having the largest case series reported by a district general hospital in England our results are in line with the one obtained in tertiary centres shows that technique is feasible and reproducible in smaller centres with appropriate training.

As the similarity of our patient groups suggests, it is reasonable to anticipate that with enhanced experience, the average intraoperative time will ultimately align with the LCR.Indeed, it has been demonstrated that RCR has a flatter learning curve with both inexperienced and experienced minimally invasive surgeons and that the operative duration advantage of LCR diminishes with increased RCR case load, such that RCR TME becomes a briefer procedure^24^. Thus, a high-volume and a standardised system of operation, may aid to further reduce the cost of robotic surgery over time^25^. This is a comparative series and, as such, no power calculations were performed to determine the numbers required to avoid type two errors and, as a non-RCT, unintentional bias may have also influenced the potential advantages demonstrated.

## Conclusions

This single-center study, representing the largest case series over two years at a district general hospital in England, investigated the non-inferiority of a robotic approach compared to laparoscopy for colorectal cancer treatment. Although the robotic data included cases from the learning curve, it demonstrated favourable surgical outcomes, including increased lymph node yield and shorter hospital stays, compared to laparoscopy. These findings suggest that robotics is a viable and effective alternative to laparoscopy for colorectal surgery, and the learning curve for experienced laparoscopic surgeons might be shallower with robotics. However, further research on the learning curve is warranted.

## Data Availability

The data that support the findings of this study are available from the first or/and the corresponding author upon reasonable request.
